# Exploring the impact of targeted distribution of free bed nets on households bed net ownership, socio-economic disparities and childhood malaria infection rates: analysis of national malaria survey data from three sub-Saharan Africa countries

**DOI:** 10.1186/1475-2875-12-245

**Published:** 2013-07-15

**Authors:** Joseph D Njau, Rob Stephenson, Manoj Menon, S Patrick Kachur, Deborah A McFarland

**Affiliations:** 1 Department of Health Policy & Management, Rollins School of Public Health (RSPH) of Emory University, 1518 Clifton Rd 16NE, Atlanta, GA, 30322, USA; 2 Hubert Department of Global Health, RSPH of Emory University, 1518 Clifton Rd 16NE, Atlanta, GA, 30322, USA; 3 School of Medicine’s Division of Medical Oncology/Fred Hutchinson Cancer Research Center, University of Washington, 1100 Fairview Ave N, MS-MI-B140, Seattle, WA, 98109, USA; 4 Malaria Branch, Division of Parasitic Disease and Malaria, at the Center for Global Health, Centers for Disease Control and Prevention (CDC), 1600 Clifton Rd, MS-A06, Atlanta, GA, 30333, USA

## Abstract

**Background:**

The last decade has witnessed increased funding for malaria control. Malaria experts have used the opportunity to advocate for rollout of such interventions as free bed nets. A free bed net distribution strategy is seen as the quickest way to improve coverage of effective malaria control tools especially among poorest communities. Evidence to support this claim is however, sparse. This study explored the effectiveness of targeted free bed net distribution strategy in achieving equity in terms of ownership and use of bed nets and also reduction of malaria prevalence among children under-five years of age.

**Methods:**

National malaria indicator survey (MIS) data from Angola, Tanzania and Uganda was used in the analysis. Hierarchical multilevel logistic regression models were used to analyse the relationship between variables of interest. Outcome variables were defined as: childhood test-confirmed malaria infections, household ownership of any mosquito net and children’s use of any mosquito nets. Marginal effects of having free bed net distribution on households with different wealth status were calculated.

**Results:**

Angolan children from wealthier households were 6.4 percentage points less likely to be parasitaemic than those in poorest households, whereas those from Tanzania and Uganda were less likely to test malaria positive by 7 and 11.6 percentage points respectively (p < 0.001). The study estimates and present results on the marginal effects based on the impact of free bed net distribution on children's malaria status given their socio-economic background. Poorest households were less likely to own a net by 21.4% in Tanzania, and 2.8% in Uganda, whereas both poorer and wealthier Angolan households almost achieved parity in bed net ownership (p < 0.001). Wealthier households had a higher margin of using nets than poorest people in both Tanzania and Uganda by 11.4% and 3.9% respectively. However, the poorest household in Angola had a 6.1% net use advantage over children in wealthier households (p < 0.001).

**Conclusion:**

This is the first study to use nationally representative data to explore inequalities in bed net ownership and related consequences on childhood malaria infection rates across different countries. While targeted distribution of free bed nets improved overall bed net ownership, it did not overcome ownership inequalities as measured by household socioeconomic status. Use of bed nets was disproportionately lower among poorest children, except for Angola where bed net use was higher among poorest households when compared to children in wealthier households. The study highlights the need for malaria control world governing bodies and policy makers to continue working on finding appropriate strategies to improve access to effective malaria control tools especially by the poorest who often times bears the brunt of malaria burden than their wealthier counterparts.

## Background

During the last decade, malaria-endemic countries have witnessed a historic increase in the amount of resources dedicated to fight the disease [1-3]. Bilateral and multilateral institutions such as the Global Fund to fight AIDS, Tuberculosis and Malaria, the World Bank and the US President’s Malaria Initiative (PMI) have more than doubled funding to help ease the burden of malaria, especially in sub-Saharan Africa (SSA) [4-7]. Additionally, non-profit private sector initiatives, such as the Bill and Melinda Gates Foundation, have played an important role in changing the debate on financing, design and implementation of malaria control programmes.

Malaria researchers and policy makers have taken advantage of heightened global malaria awareness to shift their focus to a rapid expansion of effective malaria control programmes while downplaying issues related to sustainability of these programmes. Following the increased awareness, malaria control policies, such as implementation of large-scale indoor residual spraying with insecticides and universal free bed net coverage campaigns have seen increased financial support, especially in SSA [8-11]. Meanwhile, the last 10 years has seen complete overhaul of policies addressing malaria case management in endemic settings. Most countries with high malaria burden have changed their treatment algorithms by adopting more efficacious but expensive artemisinin-based drug combinations [12-14]. Countries have also been urged to improve malaria diagnostics through adoption of universal testing of all suspected malaria cases by use of either microscopy or rapid diagnostic tests (RDTs) [14]. Support for preventive treatments in highly endemic areas through adoption of intermittent preventive treatments in pregnant women, infants and school-age children have also substantially increased [15-18]. The long-term health, economic and social impact of adopting these changes is not well understood [19,20]. However, given the positive correlation of malaria and poverty [21], it is important to understand how the current large-scale malaria control policies are impacting different segments of populations, especially the very poor.

Although initial strategies to scale up insecticide-treated nets (ITNs) relied on cost-recovery, social marketing and targeted distribution strategies (focused on biological and socio-economically vulnerable groups), increased funding has allowed for universal free bed net distributions in many SSA countries. While some countries have welcomed the new financing mechanisms and aligned their policies accordingly, some have shifted largely to respond to donor mandates alone, and others have defied the call for universal free bed net distribution as they continue with implementation of targeted bed net distribution [22,23]. Supporters of universal free bed net distribution have consistently favoured the strategy as the most feasible way to equitably reach the poor with the life-saving interventions [22,24,25]. They argue that cost sharing and targeted interventions dampen demand, enhance inequities and consequently exacerbate the malaria burden [26]. Despite their arguments, there are potential pitfalls. First, the claim that free bed net distribution enhances equity is mainly based on limited case–control studies which may be unrepresentative of real-world conditions [10,27]. As a result, such studies are not necessarily generalizable because of infrastructural and large-scale programme implementation challenges which may threaten the feasibility of reaching out to those most in need [28,29]. Secondly, given the current global fiscal austerity measures sparked by the global economic recession and the concomitant over-reliance on international development assistance, the long-term consequences of this strategy in terms of its sustainability remain uncertain [30,31]. Finally, there has been some scepticism about uniform solutions to a relatively diverse health problem and whether the disease can ever be eradicated [32-36]. Economists have also expressed concerns on the need for malaria interventions to do more by incorporating economic tenets on value for money as well as aspects of programme sustainability [37].

Therefore, any proposed solutions to African economic, socio-political and health, including those related to malaria, must first recognize and adapt to the continent’s diverse socio-economic and epidemiologic settings. Despite constituting the largest disease burden globally, malaria epidemiology in SSA varies widely [38-40]. The variations in malaria policies, strategies and epidemiology can be attributed to a number of factors, including weather and climate, altitude, physical infrastructure such as water drainage systems, level of economic development reflected in population incomes, household structures and investments in public health programmes. It is important to explore how large-scale malaria control programmes such as targeted free bed net distribution may impact malaria control efforts, especially among the poorest people. One study attempted to evaluate the health impact of a large-scale malaria control programme in Zambia [41]. However, the study did not explore how such large-scale interventions benefited various groups of people with different socio-economic backgrounds. A recent study from Malawi reported that people living closest to the health facilities were most likely to have bed nets than those living far away from health clinics [42]. Another study in Zambia reported households with a woman having attended antenatal clinic or with children under five years old were twice more likely to have bed nets than those without [29]. In Angola, people residing more than 15 km outside the capital city of Luanda were almost six times more likely to test positive for malaria when screened at the health clinic than those living in the inner-city [43]. Apart from these few studies, little is known about the impact of the large-scale implementation of malaria programmes, such as universal bed net campaigns, on household socio-economic disparities and malaria burden or access and use of effective malaria control tools.

This study aims to understand how implementation of targeted free bed net distribution has contributed to reduction of childhood malaria infection rates by their household socioeconomic disparities. The study is unique in the sense that it uses nationally representative malaria indicator survey data from three sub Saharan Africa countries of Angola, Tanzania and Uganda. It is the first study to use national data and compare inequalities in access of bed nets and their consequence in children under-five years of age malaria infection rates across the three countries with diverse malaria transmission settings and also their socioeconomic backgrounds. The study uses wealth measured as the proxy for household socio-economic status in exploring these relationships. A list of household assets including household construction materials, ownership of toilets, use of piped water at home or community sources of drinking water, furniture, and other assets like bicycles, television and sofa sets, vehicles etc. were used as detailed in MIS data collection tools [44]. More specifically, a set of malaria control indicators in children under-five defined as RDT and microscopy-confirmed positive results on the day of interview, household ownership of bed nets and children’s use of nets, will be explored and compared across districts/provinces with and without targeted free bed net distribution programs.

### Study sites and malaria control efforts

#### Angola

Despite its oil wealth and fast-growing economy, a third of Angola’s population is poor and relies on subsistence farming. Three decades of civil war ended in 2002 leaving its footprints marked by dilapidated health infrastructure, with nearly half of its total population lacking any access to health-care services [45]. In 2006 the country had one of the world’s highest under-five mortality rates estimated at 250 deaths per 1,000 live births. According to Angola’s National Malaria Control Programme, 35% of under-five mortality is attributable to malaria with stable transmission in most of the northern part of the country [46]. Following its launch in 2005, PMI embarked on supporting malaria control strategies identified by Angola’s National Malaria Control Programme. The ITN scale-up strategy in Angola consisted of both targeted free distribution and a market segmentation approach through social marketing. PMI, in collaboration with UNICEF Angola, supported targeted free ITN distribution in seven of Angola’s 18 administrative provinces. The strategy was focused on the highly malaria-endemic provinces of Cabinda, Zaire, Malanje, Moxico, Lunda Norte, Lunda Sul and Uige [47]. A total of 813,000 long-lasting ITNs were distributed to households with children under-five years during the nationwide measles immunization campaign, which occurred from July to August 2006, and also included the delivery of oral polio vaccine, vitamin A, and an anthelminthic. In addition to free net distribution, a demonstration on the proper method to hang, care for and use the ITN was provided. At least one free net was provided to each child under-five presenting for immunization in each of the seven provinces. Prior to the targeted free bed net distribution campaign, Angola had one of the lowest rates of bed net use by children under-five, estimated at less than 11% [48].

#### Tanzania

Approximately 95% of the population is estimated to be at risk of malaria, which causes between 14 and 19 million clinical episodes annually. In 2005, malaria was estimated to cause up to 36% and 20% of all deaths among children under-five and pregnant women respectively [49]. Following results from studies on efficacy and effectiveness of ITN use in Tanzania, a market-based national ITN voucher programme to scale up bed nets was adopted in 2004 [50-53]. With initial support of funding from the Global Fund, the strategy was part of nationwide malaria prevention, targeting vulnerable groups, i e, pregnant women and children under-five. Distribution of bed nets relied on the private market and existing public and private health facility infrastructure. Vouchers for ITNs were issued to women attending their first antenatal care visits at fixed price of less than US$3.00. These women used the vouchers to redeem bed nets from participating private sector bed net retailers. While overall ownership and use of bed nets through this programme improved, the coverage was generally below national and international targets. Therefore, Tanzania’s National Malaria Control Programme (NMCP) took advantage of increased international financing for malaria to implement an alternative targeted free ITN distribution strategy [53]. The new strategy was piloted by UNICEF and International Federation of Red Cross, in selected areas with high malaria burden. Targeted free distribution of bed nets to pregnant women and children under-five was implemented in the 15 districts with the lowest ITN coverage (these were: Tanga Urban and Pangani Districts in Tanga Region; Rufiji District in Coastal Region, Lindi rural, Lindi Urban, Ruangwa, Liwale, Kilwa, Nachingwea Districts from Lindi Region, and, Tandahimba, Newala, Masasi, Nanyumbu, Mtwara Rural and Mtwara Urban Districts from Mtwara Region). Over 900,000 bed nets bundled with insecticide were made available for distribution in mainland Tanzania [49,53-55].

Meanwhile in Zanzibar, a targeted free ITN distribution campaign was implemented in all 10 districts (five districts in each of the two islands of Pemba and Unguja) [56]. The Global Fund and the PMI supported the implementation of this programme, from August 2005 to early 2006. As in mainland Tanzania, the programme covered all pregnant women and children under-five. Therefore, total districts receiving targeted mass free ITN distribution in Tanzania were 25 with an estimated population of over 2.8 million people.

#### Uganda

Over 90% of the Ugandan population is at constant risk of contracting malaria. Malaria is responsible for 30-50% of all outpatient visits to health clinics and almost half of all inpatient deaths among children under-five. Annual direct malaria treatment cost for the year 2003 was estimated at $41.6 million [57]. PMI pledged to support Uganda’s NMCP strategies including a large-scale ITN distribution in the conflict districts of northern Uganda [58,59].

To improve ITN scale-up, the Ugandan NMCP adopted a mixed model approach which included: distribution of free ITN to vulnerable groups through ANC clinics and NGOs, large-scale campaigns to targeted populations and the sale of ITNs through the retail market. The strategy was complemented by annual net retreatment campaigns to ensure that ITNs maintained their effectiveness. PMI supported the distribution of ITNs to pregnant women through ANC clinics in 24 districts in northern Uganda (these were: Nebbi, Nyadri, Arua, Koboko, Yumbe, Moyo, Adjumani, Amuru, Gulu, Kitgum, Pader, Oyam, Apac, Lira, Dokolo, Amolorar, Amuria, Kaberamido, Katakwi, Abim, Kotido, Kaabong, Moroto and Nakapiripiri) [60]. By the time data collection was completed in 2009 for the Malaria Indicator Survey (MIS), over 400,000 free ITNs had been distributed to over 1.5 million people in northern Uganda. Following the bed net distribution campaign, health workers at antenatal clinics were trained and urged to explain the need to use ITNs. Health workers were also asked to demonstrate proper hanging and use of nets [59,60].

## Methods

This study is based on data from cross-sectional nationally representative MISs conducted in three SSA countries: Angola (2006), Tanzania (2007/08) and Uganda (2009). These countries were the first beneficiaries of PMI funding established in May 2005. MIS data were collected in 2006 for the first time as part of international efforts to monitor progress toward malaria control efforts in SSA. As part of these surveys, blood samples were collected and tested for both malaria parasites and anaemia in all children under-five and in self-reported pregnant women. Microscopy and/or RDTs for malaria were performed to accurately help estimate the burden of malaria in children under-five. Additionally detailed information on household ownership and use of bed nets, malaria treatment-seeking behaviours, demographic, social and economic characteristics of women of reproductive age, children under-five and a selection of men/household heads were collected [44,61].

To generate nationally representative sample sizes, MIS relied heavily on each country’s national census data to guide cluster-sampling procedures. Since malaria burden is usually thought to be higher in rural areas than in urban settings, MIS stratified these populations separately and oversampled participants from rural areas. For each rural/urban population segment, a multistage cluster sample design was implemented. A total of 1,119 eligible households from Angola, 4,340 in Tanzania and 2,296 in Uganda successfully completed the malaria indicator surveys. These constituted an overall response rate of over 95%. In all three countries, the major distribution channel for bed nets preceding MIS data collection analysed in this study, were government and faith-based health facilities. However, overtime programs have expanded to include other distribution strategies including the private sector and organised community events. To understand the varied bed net scale-up strategies adopted by each of the three countries, additional information was obtained from each country’s PMI malaria operation plans (MOPs), unpublished reports and consultation with national malaria control programme country teams.

### Ethical clearance

Prior to implementation of these surveys, IFC Micro in collaboration with each country’s local partners applied and obtained ethical clearances from respective bodies in each of the three countries as detailed in each country’s survey reports. All interview respondents consented to participating in the survey. Written informed consents were obtained from children under-five’s guardians/parents/next of kin for these surveys and publication of reports or any accompanying images compiled from the surveys [44,61].

### Dependent variables

Three main outcome variables of interest are investigated in this study. These binary outcome variables included (i) whether a child tested positive for malaria parasites on the day of the interview (positive = 1), (ii) household ownership of at least one net (yes = 1), and (iii) children’s use of bed nets in the night preceding the interview (yes = 1). For each outcome variable, separate multivariate logistic regression models with estimates of marginal effects were performed. A set of control variables relevant for each of the three equations and from each study country were included as dependant covariates.

### Independent variables

A list of important independent variables is included in the estimation model. Among these are:

i. *Place of domicile:* A dichotomous variable denoted by (1 = yes/0 = no) in reference to whether the survey respondent lived in rural area or in urban domicile.

ii. *Household wealth:* A rank variable ranging from 1 to 5 where ‘1’ represents the lowest or poorest quintile and ‘5’ represented the wealthiest household was used. The 1st and 2nd poorest households were collapsed into one group denoted by 0 to represent the poorest households whereas the top quintiles 3rd to 5th groups were collapsed to a single value to represent wealthier households. The ranking of household wealth was based on household assets index scores through principal component analysis (PCA) as developed by Filmer D and Pritchett L [62].

iii. *Access to media:* A binary variable (yes = 1) reporting ownership and use of a television set, radio or reading newspapers at least once a week was used to represent individual’s access to media.

iv. *Malaria endemicity:* An ordinal variable indicating regional level malaria transmission status in terms of altitude and malaria endemicity as recorded by international malaria atlas (MARA) project [40]. The variable ranges from 0 to 2 with 0 representing low malaria transmission, 1 for moderate transmission and 2 for high malaria transmission levels.

v. *Bed net ownership:* A binary variable (yes = 1) indicating whether or not a household possessed any bed net regardless of whether it was long-lasting insecticide-treated net (LLIN) or any other net. *(vi) Other variables include;* number of children under five years of age living within a given household, size of the household in terms of total number of people living in, proximity to health facility measured by estimated kilometre distance, age, gender and education level of household head as well as interaction term indicating whether or not, the region had large scale free bed-net distribution campaign.

### Empirical analysis

Since the MIS data has a hierarchical structure, the central assumption of linear independence in ordinary logistic regression models is violated. To address this particular problem, the analysis uses a multilevel modelling technique to account for both the hierarchical structure and to allow the identification of individual and community level influences on the outcomes [63]. Practically, malaria policies and strategies as well as epidemiology vary across countries and across regions. Within a given country, these variations depend on a number of factors such as weather, climate, altitude, physical infrastructure including water drainage systems, level of economic development reflected by the population’s incomes, household structure as well as investments in public health programs. Therefore, cluster analysis is the most appropriate strategy to address these intra-regional/country variations. Additionally, multilevel modelling enables efficient estimates of parameters with corrected standard errors and allows for clustering of observations within units [64]. In this study, the estimation strategy provides a measure of the extent to which the odds of a child being malaria positive, a household owning a net and reported child’s use of a net vary across different cluster units. Based on these estimates, the probabilities for each dependent variable were modelled and the calculation of their marginal effects performed using the STATA software package, version 11 (Stata Corp., College Station, Tx, USA).

Based on past studies a list of household and community/country level explanatory variables expected to correlate with the study’s outcome variables were included in each of the estimation models. Additionally, pair-wise correlation analyses between dependent variables and relevant covariates were performed to help determine the most relevant independent variables for inclusion. For this analysis, a three tier-level of important variables were included. These are: (i) individual level demographic characteristics such as age, education, gender and marital status; (ii) household level characteristics such as wealth, place of domicile, proximity to health facility and the level of media exposure household members enjoy; and, (iii) community, regional and country level characteristics, such as regional malaria transmission intensity, administrative divisions and existence of targeted free bed net distribution programme.

### Interaction terms

This constitutes interaction between wealth variable and existence of any bed net distribution campaigns within a given community. This is expected to capture the relationship between household wealth status and any existing package of free distribution of bed nets to households aimed at protecting household members from malaria disease. Since the analysis uses logistic regression models, the study further estimated marginal effects for each of the important control variables [65,66]. Marginal effects of having targeted free bed net distribution by each respective outcome variables of interest in relation to households’ wealth status were predicted. Finally, the study explored a scenario for not having free bed net distribution given the wealth status of the households and their impact on outcome variables.

Therefore, the general form of the random intercepts multilevel logistic regression model for each of our three outcome variables may be expressed as follows:

(1) $$\begin{array}{l} \mathrm{Logit}\;M_{1\mathrm{ijk}}=\mathrm{SE}{S^{\prime }}_{1\mathrm{ijk}}\;\beta_{11}+{X^{\prime }}_{\mathrm{ijk}}\beta_{12}+u_{1\mathrm{jk}}\\ \;+\nu_{1k} \end{array}$$

(2) $$\begin{array}{l} \mathrm{Logit}\;N_{2\mathrm{ijk}}=\mathrm{SE}{S^{\prime }}_{2\mathrm{ijk}}\;\beta_{21}+{X^{\prime }}_{2\mathrm{ijk}}\;\beta_{22}+u_{2\mathrm{jk}}\\ \;+\nu_{2k} \end{array}$$

(3) $$\begin{array}{l} \mathrm{Logit}\;E_{3\mathrm{ijk}}=\mathrm{SE}{S^{\prime }}_{3\mathrm{ijk}}\;\beta_{31}+{X^{\prime }}_{3\mathrm{ijk}}\;\beta_{32}+u_{3\mathrm{jk}}\\ \;+\nu_{3k} \end{array}$$

where *M*_*1ijk*_ is the probability of a child *i*, in *j*^*th*^ household and administrative region *k*, being malaria positive on the day the survey was conducted; *SES’*_*ijk*_ is a composite of household socio-economic disparities (education level of the household head, household wealth and living in urban or rural domicile); *X’*_*ijk*_ is a vector of other independent covariates including the interaction terms between malaria programme implementation and household socio-economic status. The *β’s* are associated vector of regression parameter estimates and *u*_*jk*_ and *v*_*k*_ are the residuals at respective household and administrative region levels. Other outcomes: *N*_*2ijk*_ in equation (2) captures the probability of a household owning at least one bed net of any type and, *E*_*3ijk*_ in equation (3) represents the probability of at least one child within the household sleeping under any bed net on the night before the survey was conducted. All residuals are assumed to be independent, normal and homoscedastic (i e, zero mean and constant variances) [67,68].

## Results

The majority of surveyed households in Tanzania and Uganda were from rural areas, 82% and 86% respectively, whereas in Angola only 51% of households were from rural areas. The average household size was roughly the same across the three countries at 5.1 in Angola, 5.3 in Tanzania and 4.8 in Uganda. Heads of households reporting no formal education ranged from 21% in Uganda and 28% in Tanzania to 30% in Angola. Female-headed households in all three countries were less than 30%, with Uganda the highest at 29% (Table 1).

**Table 1:** Descriptive statistics from cross-sectional national Malaria Indicator Surveys for children aged six to 59 months

**Key outcome and control variables**	**Angola (1,119)**	**Tanzania (4,340)**	**Uganda (2,296)**
Year of the national MIS	2006/07	2007/08	2009
***Some basic household characteristics***			
Households living in rural areas	571 (51%)	3559 (82%)	1974 (86%)
Children with confirmed malaria infections	214 (20%)	782 (18%)	895 (39%)
Overall households with bed nets	369 (33%)	2,952 (68%)	1,400 (61%)
Households reporting children’s use of bed nets	246 (22%)	1,650 (38%)	941 (41%)
Female-headed households	243 (22%)	765 (24%)	666 (29%)
Average household age	44 Years	45.8 Years	41.47 Years
Average household size	5.1	5.33	4.82
Average household number of children <5	1.87	1.69	1.11
Household with no media access	NA	1,786 (41.2%)	1,107 (48.2%)
Households residing over 3kms away from the closest health facilities	NA	1,883 (43.4%)	1,458 (63.5%)
***Household heads education levels***			
No formal education	336 (31%)	1,216 (30%)	482 (22%)
Some Primary, Secondary or College education	772 (69%)	3,039 (70%0	1,791 (78%)
***Household wealth category***			
Poorest	492(44%)	1650 (38%)	918 (40%)
Wealthier	615(56%)	2,648 (62%)	1354 (60%)
***Households living in areas receiving free bed-net distribution (FBD)***			
Households from areas receiving targeted FBD	**515 (46%)**	**1,387 (32%)**	**918 (40%)**
Proportion of children with malaria in FBD	109 (21%)	412 (26%)	242 (33%)
Households with bed nets in area receiving FBD	206 (40%)	1000 (63%)	477 (65%)
Children using bed nets in area receiving FBD	109 (21%)	665 (42%)	344 (46%)

Households reporting ownership of at least one bed net was 33% in Angola, 61% in Uganda and 68% in Tanzania. Overall bed net use by children under-five was highest in Uganda at 41% and lowest in Angola at 22%.

### Childhood malaria infection rates

**Figure 1:**
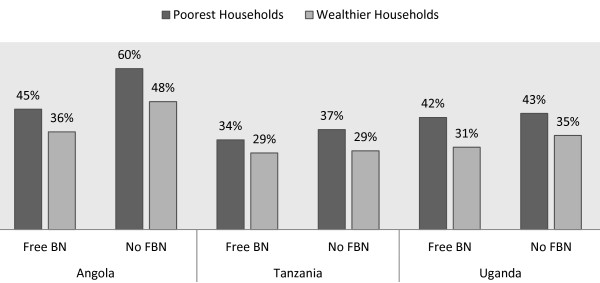
Children’s malaria infection by household wealth and by bed net distribution strategy.

Of the three countries, only Uganda used both RDT and microscopy to confirm malaria parasitaemia in children under-five. For our results to be comparable, we only used RDT test results for Uganda in order to be able to compare these with those from Angola and Tanzania which were also based on Paracheck Pf™ RDT for malaria in children under-five years of age. Malaria confirmed cases in children under-five were lowest in Tanzania at 18% and highest rate was recorded in Uganda, 39%. Irrespective of the level of household bed net ownership levels or any country-specific characteristics, household wealth was strongly correlated with malaria-positive results in children under-five (Table 2). Other important covariates with at least 10% or less of statistical significance to outcome variable included: the size of the household in terms of number of people living within the household, bed net ownership, education level and gender of the household head, urban *versus* rural location, proximity to any formal health facility and regional variation in malaria endemicity (Figure 1).

**Table 2:** Multilevel model results with an outcome of RDT confirmed malaria parasitaemia in children

**Total observations (N)**	**Angola** **1,125**		**Tanzania** **3,109**		**Uganda** **1,954**	
***Children’s RDT confirmed malaria***	***mfx dy/dx***	**Confidence intervals**	***mfx dy/dx***	**Confidence intervals**	***mfx dy/dx***	**Confidence intervals**
Age of household head	−0.009	−0.0012 – 0.0032	0.006	−0.0004 – 0.0016	0.001	−0.0005 – 0.0029
Gender (Male-headed households)	−0.023	−0.0977 – 0.0502	−0.029*	−0.0637 – 0.0049	0.010	−0.0381 – 0.0690
Education of household head	−0.009	−0.0776 – 0.0593	0.027*	−0.0023 – 0.0567	0.015	−0.0386 – 0.0690
Place of domicile	0.022**	−0.0781 – 0.1228	0.055*	0.0005 – 0.1097	0.050	−0.1701 – 0.0688
Household wealth	−0.034***	−0.1543 – 0.0773	−0.070***	−0.0943 – 0.0267	−0.116***	−0.1876 - -0.0583
Malaria endemicity	0.010*	−0.0778 – 0.0572	0.095***	0.0357 – 0.1561	0.288**	−0.5526 - -0.0247
Size of the household	0.015*	0.0021 – 0.0285	0.004	−0.0059 – 0.0050	0.005	−0.0163 – 0.0055
Number of children aged <5 years	−0.025**	−0.1787 – -0.0181	0.049***	0.0331 – 0.6565	−0.044**	−0.0742 - -0.0156
Ownership and use of any bed net	−0.055**	−0.1187 – 0.0083	−0.034*	−0.1233 – 0.0387	−0.098***	−0.0419 – 0.1494
Access to media	NA	NA	−0.016	−0.0425 – 0.0094	−0.034	−0.0149 – 0.0848
Proximity to health facilities	NA	NA	0.084***	0.0560 – 0.1128	0.102***	0.0525 – 0.1521
Existence of free bed-net distribution	0.251	0.0226 – 0.4801	−0.015*	−0.0134 – 0.0405	−0.082**	0.1479 – 0.0494
Interaction term (free bed net/wealth)	−0.046**	−0.0668 – 0.1772	−0.009	−0.0612 – 0.0415	−0.064	−0.0393 – 0.1690
***Predicted marginal effects for free bed-net distributions and wealth with reported positive malaria case in children***						
	**Angola**		**Tanzania**		**Uganda**	
*Dependent variable:****Malaria***	*Marginal effects: (dy/dx)*		*Marginal effects: (dy/dx)*		*Marginal effects: (dy/dx)*	
1 Wealth	−0.034*		−0.070***		−0.116***	
2 Free bed-net distribution	0.251*		−0.015		−0.081**	
3 Scenarios for interaction term (free bed nets/wealth)						
No free bed nets in poorer households	0.190***		0.257***		0.414***	
No free bed nets in wealthier households	0.168***		0.187***		0.291***	
Free bed nets in poorer	0.476***		0.272***		0.323***	
households						
Free bed nets in wealthier households	0.431***		0.199***		0.218***	

Note: * statistically significant at 10% level, ** significant at 5% level and, *** refers to significance level equal to or, less than 1%.

Following results from the multilevel regression models, a prediction of the probability of a child being infected given its household wealth status and other covariates was performed. The marginal effects for a child to be malaria test positive if he/she belonged to wealthier household was reduced by 3.4, 7 and 11.6 percentage points for among children in Angola, Tanzania and Uganda respectively (all at p < 0.001). Additionally, the number of people living within a household in Angola was positively correlated with positive childhood malaria cases. The predicted marginal effects of size of households for malaria positive children in Angola was 1.5 percentage points (p < 0.05) but was insignificant in both Tanzania and Uganda. Meanwhile marginal effects for bed net ownership showed a moderate reduction of childhood malaria positive cases. A 5.5 percentage points reductions in Angola was recorded (p < 0.05), 3.4 percentage points in Tanzania (p < 0.10) whereas, Uganda exhibited the largest reduction of childhood malaria infection rates at 9.8 percentage points (p < 0.001). Households living far away from health clinics (over 3 km in Uganda and Tanzania) had their children reporting significant higher rates of malaria positive than those living closer to health facilities. The predicted marginal effects for children being malaria positive showed an increase of 8.4 and 10.2 percentage points in Tanzania and Uganda respectively (p < 0.001). Unfortunately, same data on household proximity to health facilities were not available for Angola.

Furthermore, an exploration on whether targeted free bed-net distribution policy had any impact on reported malaria positive cases in children in all three countries was performed. The marginal effects for free bed net distribution on malaria parasites indicated negative association in both Tanzania and Uganda at 5% and 10% significant level. However, only Uganda exhibited substantial reductions of 8.2 percentage points. Despite targeted free bed net distribution, predicted marginal effects estimated that children in poorest households in all three countries were more susceptible to malaria than children from wealthier households. Across the three countries, children from wealthier households were less likely to test malaria positive by 4.5% in Angola, 7% in Tanzania and by 10.5% in Uganda (p < 0.001).

### Ownership of any mosquito net

**Figure 2:**
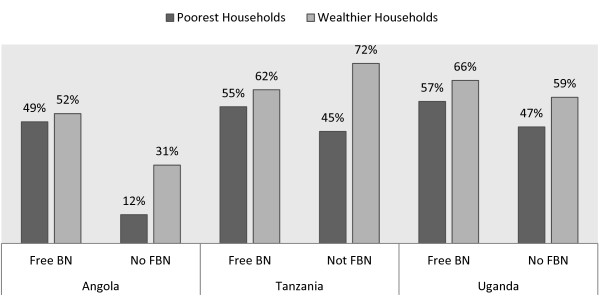
Bed net ownership by household wealth and by distribution strategy.

Education and gender of the household head as well as the urban *versus* rural location had a varied degree of significant relationship with household ownership of any bed nets across the three countries. Household wealth exhibited by far the strongest association with ownership of bed nets (Figure 2). The predicted marginal effect for households in rural Angola to have bed nets was indicated a 2 percentage point less likely to own any bed nets (p < 0.10). In Uganda, rural households were 13.3 percentage points less likely to own bed nets. Education and gender of the household head were other significant covariates for household ownership of any bed nets in Uganda and Tanzania (Table 3). Male-headed households were less likely to own nets by up to 5.3 percentage points in Uganda (p < 0.01). Age of the household head, number of children under-five and the size of the household were other important variables exhibiting significant relationship with household bed net ownership. About 46% of households in Angola were from seven provinces, which had received targeted free bed net distribution whereas for Tanzania and Uganda such households were 32% and 40% respectively (Table 1). Of all three-study countries, the dummy variable for implementation of targeted free bed net distribution showed a significant relationship with households’ bed net ownership in Angola only. Nevertheless, the exploration of different scenarios of bed net distribution and calculation of their marginal effects are reported (Table 3). Targeted free bed net distribution achieved 52 percentage points of bed net coverage for both poorest and wealthier households in Angola (p < 0.001). In Tanzania, free bed net distribution was unable to bridge the 21% gap of bed net ownership between poorest and wealthier households, whereas in Uganda, bed net ownership gap between poorest and wealthier households was narrowed down a difference of to about 3% only (p < 0.001).

**Table 3:** Multilevel model results for household ownership of bed nets

**Total observations (N)**	**Angola** **1,125**		**Tanzania** **3,109**		**Uganda** **1,954**	
***Household ownership of any bed net***	***mfx dy/dx***	**Confidence intervals**	***mfx dy/dx***	**Confidence intervals**	***mfx dy/dx***	**Confidence intervals**
Age of household head	−0.031*	−0.0058 - –0.0005	−0.022***	−0.0034 - –0.0010	−0.017*	−0.0034 - –0.0002
Gender (Male-headed households)	−0.022	−0.1058 – 0.0616	−0.038**	−0.0760 - -0.0017	−0.053**	−0.10144 - -0.0061
Education of household head	0.056	−0.1365 – 0.0226	0.040*	0.0045 – 0.0762	0.046*	−0.0087 – 0.10154
Place of domicile	−0.020	−0.0967 – 0.1384	−0.019	−0.0656 – 0.0276	−0.133*	0.01417 – 0.2520
Household wealth	0.084**	−0.1318 – 0.1386	0.152***	0.1134 – 0.1910	0.120***	0.05314 – 0.18817
Malaria endemicity	0.028	−0.0509 – 0.1074	−0.019	−0.0747 – 0.0359	0.041	−0.1910 – 0.27487
Size of the household	0.017*	0.0029 – 0.0327	0.005*	−0.0009 – 0.01198	−0.005	−0.0114 – 0.01025
Number of children aged <5 years	0.020	−0.0497 – 0.0091	0.022**	0.0036 – 0.04060	0.006	0.0219 – 0.03487
Malaria parasitaemia	−0.068*	−0.1451 – 0.0082	−0.017	−0.0163 – 0.0519	−0.099***	−0.14765 -0.05042
Access to media	NA	NA	0.011*	−0.01860 – 0.0411	0.035	0.08464 – 0.01384
Proximity to health facilities	NA	NA	−0.200***	−0.2305 - -0.1701	0.002	0.04486 – 0.48889
Existence of free bed-net distribution	0.023*	−0.2408 – 0.2876	0.025	−0.0834 – 0.0270	0.083	−0.30870 – 0.14176
Interaction term (free bed net/wealth)	0.055*	−0.0781 – 0.1883	0.005	−0.0646 – 0.0539	0.002	−0.09614 – 0.09659
***Predicted marginal effects for free bed-net distributions and wealth with reported household ownership of any bed net***						
	**Angola**		**Tanzania**		**Uganda**	
*Dependent variable:****Household bed-net ownership***	*Marginal effects: (dy/dx)*		*Marginal effects: (dy/dx)*		*Marginal effects: (dy/dx)*	
1 Wealth	0.084**		0.152***		0.120***	
2 Free bed-net distribution	0.023*		0.025		0.083	
3 Scenarios for interaction term (free bed nets/wealth)						
No free bed nets in poorer households	0.297***		0.582***		0.552***	
Free bed nets in poorer households	0.520***		0.548***		0.656***	
Free bed nets in wealthier households	0.523***		0.762***		0.684***	

Note: * statistically significant at 10% level, ** significant at 5% level and, *** refers significance level equal to or, less than 1%.

### Bed net use among children under the age of five years

**Figure 3:**
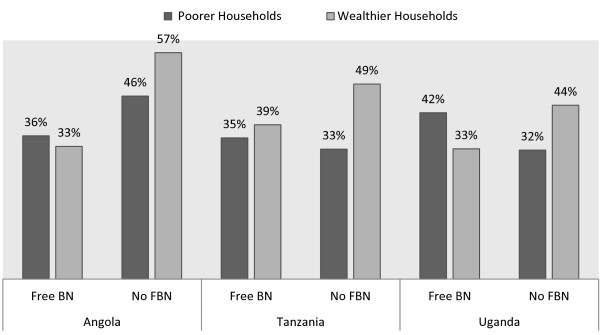
Bed net use by household wealth and by distribution strategy.

Household wealth, age, education of the household head, number of children under-five, malaria endemicity and proximity to health facilities all exhibited some degree of significant relationship with children’s use of bed nets. Overall, household wealth was the strongest predictor of children’s use of bed nets across the three countries (Figure 3). Wealth had the strongest impact on children’s bed-net use in Tanzania with marginal effects estimated at 17.7 percentage points whereas in Angola and Uganda the predicted marginal effects were 6 and 13.7 percentage points respectively. The higher the number of children reported to be living within a household, the better the chances for them to sleep under a bed net in at least Tanzania and Uganda. The chances for children to use bed nets increased with the total number of children under-five years of age living within a household by the respective 3.7 and 4.8 percentage points in both Tanzania and Uganda (Table 4). The marginal effects estimates on the impact of free bed net distribution strategy on bed net use by children under the age of five years were found to be significant only in Angola and Tanzania. The study tested for the impact of free bed net distribution on their usage among poorest and wealthier households. These results showed that free bed net distribution improved their usage among poor households in Angola by almost 28% with a 6% advantage over children in wealthier households (p < 0.001). For Tanzania and Uganda, the chances of increased bed net use by children in poorer households due to free bed net distribution strategy were not improved. Children in wealthier households in both Tanzania and Uganda continued to have upper hand on bed net use by 11% and 4% respectively when compared to those from poorest households (p < 0.001).

**Table 4:** Multilevel model results for children’s use of bed nets

**Total observations (N)**	**ANGOLA** **1,125**		**TANZANIA** **3,109**		**UGANDA** **1,954**	
***Children’s use of any bed net***	***mfx dy/dx***	**Confidence intervals**	***mfx dy/dx***	**Confidence intervals**	***mfx dy/dx***	**Confidence intervals**
Age of household head	−0.03	−0.0016 – 0.0023	−0.018**	−0.0031 - –0.0004	−0.045***	−0.0065 - –0.0025
Gender (Male-headed households)	−0.015	−0.0653 – 0.0622	−0.014	−0.0565 – 0.0280	−0.052*	−0.1064 – 0.0021
Education of household head	0.039*	−0.0188 – 0.0970	0.026	−0.0134 – 0.0662	0.004	−0.0547 – 0.0640
Place of domicile	0.062	−0.0227 – 0.1484	−0.030	−0.0779 – 0.1778	0.137*	0.0241 – 0.2515
Household wealth	0.060**	−0.6844 – 0.1260	0.177***	0.1379 – 0.2178	0.137***	0.0649 – 0.2098
Malaria endemicity	−0.130***	−0.1897 - -0.0707	−0.010	−0.0660 – 0.0457	0.050	0.1747 – 0.2749
Size of the household	−0.008*	−0.0020 – 0.0966	−0.005	0.0125 – 0.0015	−0.013*	−0.0252 - -0.0008
Number of children aged <5 years	−0.011	−0.0357 – 0.0118	0.037***	0.0168 – 0.05725	0.048**	0.01680 – 0.12906
Malaria parasitaemia	−0.041	−0.09667 – 0.0135	−0.011	−0.02726 – 0.0505	−0.147***	−0.19874 - -0.0972
Access to media	NA	NA	0.012*	−0.0450 – 0.0200	0.002	−0.05523 – 0.0558
Proximity to health facilities	NA	NA	−0.199**	−0.2303 – 0.1677	−0.032	−0.0322 – 0.07353
Existence of free bed-net distribution	0.019*	−0.06567 – 0.1041	0.053*	0.1152 – 0.0075	0.029	0.2382 – 0.20291
Interaction term (free bed net/wealth)	0.009	−0.09466 – 0.1130	0.036	−0.0324 – 0.10533	0.075	−0.1816 – 0.03141
***Predicted marginal effects for free bed-net distributions and wealth with reported children’s use of bed nets***						
	Angola		Tanzania		Uganda	
*Dependent variable:****Children’s bed net usage***	*Marginal effects: (dy/dx)*		*Marginal effects: (dy/dx)*		*Marginal effects: (dy/dx)*	
1 Wealth	0.060**		0.177***		0.137**	
2 Free bed-net distribution	0.091*		0.053**		−0.029	
3 Scenarios for the interaction term (free bed nets/wealth):						
No free bed nets in poorer households	0.270***		0.197***		0.237***	
No free bed nets in wealthier households	0.209***		0.314***		0.279***	
Free bed nets in poorer households	0.279***		0.189***		0.210***	
Free bed nets in wealthier households	0.218***		0.303***		0.249***	

Note: * statistically significant at 10% level, ** significant at 5% level and, *** refers to significance level equal to or, less than 1%.

## Discussion

The study results show that malaria test positive cases were proportionately higher in children from the poorest households than those from wealthier households. While there was slight reduction in the number of children with positive malaria tests in areas with free bed net distribution, malaria infection remained concentrated in children from poorest households. Marginal effects estimates showed that children from wealthier households were up to 12% less likely to have malaria parasites than those in poorest households. In terms of bed net ownership, belonging to a wealthier household increased the probability of net ownership by 3% for those living in Angola, 21% in Tanzania and 12% in Uganda. Free bed net distribution contributed to a substantial increase of bed net ownership among the poorest households in Angola as they achieved parity with wealthier households. Clearly for Angola, ownership of any bed net was significantly higher in provinces with free distribution than in provinces without a targeted distribution. Poorest households in provinces with free bed net provision had a 21% advantage over those in areas without the strategy. For both Tanzania and Uganda, there was overall improvement in bed net ownership, yet poorest households had proportionately lower probability of reporting bed net ownership than wealthier households. Regarding children’s use of bed nets, free bed net distribution did not improve usage in all three countries. In Tanzania and Uganda for instance, children from poorest households living in areas with free bed net distribution were significantly less likely to use the nets by 1% and 3% respectively, than those in wealthier households. Moreover, with the exception of Angola where children in poorest households had a 6.1% advantage of net use over those from wealthier households, children in the poorest households were less likely to use bed nets in Tanzania and Uganda by 11.4% and 3.9% respectively compared to their wealthier counterparts.

This analysis corroborates with other studies to confirm that malaria burden is often concentrated on economically poor households [21,69]. Studies conducted in Kenya and Togo established this socio-economic phenomenon whereby children in poor households and with poor nutritional status exhibited a greater risk of having high-density malaria parasitaemia, clinical malaria and severe anaemia than those in wealthier households or non-stunted children [70,71]. Children living in poorest households are therefore more vulnerable to malaria than those living in wealthier households. In terms of household bed net ownership, the findings indicate that targeted free bed net distribution improved overall coverage but did not achieve parity across households with different wealth status. Other studies have also reported unequal distribution of bed nets across countries in SSA [72-76]. Despite large-scale implementation of targeted free bed net distribution, unequal access to bed nets across households remained relatively high especially in Tanzania and Uganda. Inequities in access to these life-saving malaria interventions could partly be attributed to the type of distribution channels chosen by each country and, to the high degree of variation in physical infrastructure, which inhibited effective bed net delivery mechanisms in some settings. In places like Angola where almost 80% of the health facility infrastructures were destroyed by civil wars, the success of such a strategy becomes even more daunting. The same can be said for northern Uganda where civil wars have ravaged the region for over 20 years debilitating most of the regions’ physical infrastructure to effective delivery of public health programmes, such as bed net distribution. Moreover, studies have also found that the poorest people often live far away from health clinics, which contributes to their inability to access bed nets when distributed through such channels [77-80].

This current analysis was based on three malaria indicator surveys from Angola, Tanzania and Uganda. These surveys were conducted at the time when all three countries embraced targeted bed net distribution as an effective strategy for scaling up bed net coverage for malaria control. However, this analysis shows that targeted strategies failed to overcome bed net ownership inequities, a fact which was realized by most malaria-endemic countries as well as global malaria control programme donors [10,22,81]. Following this realization, mass universal campaigns have been conducted across many countries as a ‘catch up’ strategy to address these inequities. To a larger extent, there have been huge gains in terms of increasing bed net coverage especially over the last five years [25,82,83]. Despite these gains, it is important to note that most countries have still continued to rely on targeted bed net distribution as a strategy for their ‘keep up’ [56]. It is therefore important to conduct further analysis on more recent MIS data to explore whether the trend exhibited in this study is being reversed as some scholars have argued for increased universal free bed net coverage [81,84].

Finally, these findings on low bed net use among children under-five of all socio-economic backgrounds underscore the need for better understanding of some of the important determinants of bed net use across households with different socio-economic status. A study conducted in two districts in Tanzania found that in one district household wealth was a major determinant factor for ITN use but in another district ITN use was not tied to wealth of the household. Furthermore, the same study found a near-perfect equality in bed net use in the second district compared to the first district in which wealthier households were more likely to use ITNs than poorest households [85,86]. In another study completed in Western Kenya highlands, the authors reported that bed net use was positively correlated with education level of the household head [87]. A study in Uganda attributed low bed net use among biologically vulnerable groups to low sensitization and education on proper use from health workers and other bed net distributing agencies [88]. These studies showcase our limited understanding of the factors determining ownership and use of these important malaria control tools [89-93].

There are two important limitations of this study worth discussing. First, the study used cross-sectional malaria survey data from all three countries. With cross-sectional data it is virtually impossible to assign causal attributes between variables. Therefore, this study can only point to the evidence indicating existence of some form of association between the dependent and independent variables of interest but cannot confidently infer any causal relationships. Secondly, the survey’s sampling procedure deliberately oversampled rural households emphasizing the fact that malaria disease is predominantly a rural problem. This has resulted in lower statistical power in estimating the impact of childhood malaria prevalence and household ownership and use of bed nets in urban areas. The sampling strategy undermines the fact that poorer households in urban settings can sometimes experience worse living conditions than those living in rural places.

### Policy implications

This study has shown that targeted free bed net distribution policy in itself may not address the problem of unequal access to these life-saving interventions. Despite some improvements in net ownership, targeted free bed net distribution strategy as described here did not address the chronic problem of disproportional access to bed nets especially among poor households. The findings suggest that there are other non-financial factors that may be contributing to unequal bed net ownership within households across countries. Structural bottlenecks may play an important role in limiting poor people from accessing these effective interventions. It is therefore important to understand the other limiting factors; otherwise policies such as the universal free bed net distribution recently endorsed by the WHO are unlikely to achieve the desired outcomes. Moreover, with the exception of Angola, targeted bed net distribution did not increase poorest households’ children’s bed net usage. Children living in poorest households with nets in Tanzania and Uganda reported less use of the bed nets than those living in relatively wealthier households. A number of studies have suggested factors such as sleeping arrangements, sleeping structures and mobility as some of the reasons for poor net use [94-97]. Increased resources for targeted or, mass free bed net distribution may not necessarily improve use of bed nets especially among poorest households. However, it will be intriguing to see how this plays out in future surveys given the recent increased resources and support for universal free-bed net distribution.

Finally, individuals residing in rural areas continued to disproportionately own and use less bed nets than their urban counterparts. Historically malaria has been shown to be a predominant problem especially in rural areas. With increased urbanization and improved urban infrastructures, perhaps it is important for malaria policy makers to concentrate more on designing strategies that will increase ownership and use of bed nets in rural areas. The rural infrastructure in terms of better roads and housing may also be more challenging and, unless special efforts are put in place for better improvement of overall rural lives; efforts to reduce malaria in rural places may be substantially limited. Since the majority of people in sub Saharan Africa continue to live in rural areas, malaria policy makers should prioritize malaria control efforts in these places.

## Conclusions

This is the first study to use nationally representative data from three countries in sub Saharan Africa to explore inequities in bed net distribution and use and their consequences in reducing malaria infections in children aged under-five years. The study has shown that implementation of targeted free bed net distribution in Angola, Tanzania and Uganda did achieve an overall improvement in bed-net coverage across households with different socioeconomic statuses. However, these improvements were not equitable and did not match the substantial multilateral and bilateral donor support these countries enjoyed for a period of over four years. The relationship between targeted distribution of free bed nets and reduction of childhood malaria infections, ownership and use of bed nets among poorest households was less significant relative to wealthier households. For instance, with the exception of Angola; targeted free bed net distribution did not exhibit any improvements in bed net use among children under-five living in poorest households in Tanzania and Uganda.

The fact that targeted free distribution of bed nets did not eliminate the structural bottlenecks that inhibit poorest households from accessing effective malaria control interventions like bed nets poses some challenges to malaria experts. It emphasizes the need to critically evaluate the current malaria control strategies to establish their effectiveness especially when implemented on large scale. Given the diverse institutional and infrastructural challenges facing many SSA countries, it is not surprising that wealthier households in some settings would potentially benefit more from targeted free bed net distribution programmes than poorest households. Malaria experts should continue to be engaged in designing better and more effective malaria control policies in terms of improving their access especially by the poorest. While universal bed net distribution has been strongly endorsed by world health governing bodies the policy may not necessarily achieve equitable access unless deliberate strategies are put into place to ensure this objective is achieved.

### Disclaimer

The findings and opinions expressed by authors in this article do not necessarily reflect the opinion of the Centers for Disease Control and Prevention or the institutions with which the authors are affiliated.

## Competing interests

All authors of this article declare that they have no competing interests.

## Authors’ contributions

JDN conceived the study. SPK, MM, DAM and RS contributed ideas to improve on the study and suggested appropriate analytical strategies. JDN, MM and SPK requested access to MIS data from ICF Macro. JDN analysed the data and produced the first draft of the paper. All authors read and contributed to the development of the final draft of the paper. All authors approved the final draft in its current format.
